# Minimally invasive screw fixation versus conservative treatment for geriatric non- or minimally displaced acetabular fractures: a retrospective cohort study

**DOI:** 10.1007/s00590-026-04826-7

**Published:** 2026-06-15

**Authors:** Jana Mayer, Alexander Eickhoff, Carlos Pankratz, Florian Gebhard, Raffael Cintean

**Affiliations:** 1 University of Ulm, Ulm, Germany; 2 Department for Orthopaedic Trauma, University Hospital Ulm, Ulm, Germany

**Keywords:** Acetabular fractures, Geriatric, Minimalinvasive, Navigation, Mobilization

## Abstract

**Background:**

Acetabular fractures, though rare, present significant challenges due to complex anatomy and patient comorbidities, particularly in the elderly. While open reduction and internal fixation remain standard for most fractures, they are associated with high perioperative morbidity, especially in the elderly. Minimally invasive screw fixation may allow early mobilization while avoiding the morbidity of open reduction. The aim of this study was to compare clinical outcomes between operative minimally invasive fixation and conservative treatment in geriatric patients with non- or minimally displaced acetabular fractures.

**Materials and methods:**

This retrospective cohort study included geriatric patients with non- or minimally displaced acetabular fractures treated between 2015 and 2023 at a level I trauma center. Patients underwent either minimally invasive screw fixation or conservative treatment. The primary outcome was in-hospital mobility loss, defined as the difference between pre-injury Charité Mobility Index (CHARMI) and the best CHARMI score achieved during hospitalization. Secondary exploratory outcomes included in-hospital medical complications, loss of autonomy, length of hospital stay, conversion to surgery, and one-year mortality. Group comparisons were performed using Fisher’s exact test and Welch’s t-test. Multivariable regression analyses were performed using predefined clinically relevant covariates.

**Results:**

A total of 151 patients were included, comprising 92 operatively treated and 59 conservatively treated patients. Baseline characteristics were comparable between groups. In unadjusted analysis, operatively treated patients showed less in-hospital mobility loss than conservatively treated patients (CHARMI difference 2.53 ± 1.73 vs. 3.44 ± 1.57; mean difference − 0.91, 95% CI − 1.45 to − 0.37; *p* = 0.0011). However, after adjustment for baseline mobility and other clinically relevant confounders, treatment modality was not independently associated with mobility loss (β − 0.07, 95% CI − 0.59 to 0.44; *p* = 0.780). Secondary exploratory analyses showed lower one-year mortality in the operative group in unadjusted analysis (10.9% vs. 30.5%; OR 0.28, 95% CI 0.12–0.66; *p* = 0.0046), but this association was not confirmed after multivariable adjustment. Treatment modality was also not independently associated with loss of autonomy, medical complications, or length of hospital stay.

**Conclusion:**

In this retrospective cohort, minimally invasive screw fixation was associated with less in-hospital mobility loss in unadjusted analysis, but this association was not confirmed after adjustment for baseline mobility and other confounders. Mortality and other secondary outcomes should be interpreted as exploratory. Minimally invasive fixation may be considered in selected patients who fail to mobilize adequately under conservative treatment, but no causal conclusion regarding superiority over conservative management can be drawn.

## Introduction

Acetabular fractures are relatively uncommon injuries, accounting for approximately 3–8% of all fractures. Despite their low incidence, they are associated with substantial morbidity and mortality and pose considerable challenges for both surgical treatment and postoperative care [1–4]. Traditionally, acetabular fractures were primarily associated with high-energy trauma in younger patients. However, demographic changes have led to a steadily increasing incidence of osteoporotic pelvic and acetabular fractures in the elderly population [4–6].

The management of acetabular fractures in geriatric patients is particularly demanding. Many patients present with multiple comorbidities and reduced physiological reserves, which significantly increase perioperative risk [5]. Open reduction and internal fixation remains the standard treatment for displaced fractures but is associated with substantial surgical trauma and potentially high complication rates, especially in elderly and frail patients [7].

Minimally invasive screw fixation has therefore gained increasing attention as a potential alternative in selected fracture patterns. Percutaneous stabilization allows fracture fixation while minimizing surgical exposure and soft-tissue trauma [8]. Recent advances in intraoperative imaging and computer-assisted navigation have further improved the precision of screw placement, which is particularly important in the complex anatomy of the acetabulum [9–11].

For non- or minimally displaced acetabular fractures, treatment decisions remain controversial. Conservative management is frequently chosen and may include early mobilization with weight-bearing as tolerated using assistive devices in selected patients. However, in clinical practice, mobilization may still be limited by pain, frailty, concerns regarding fracture stability, or limited ability to comply with individualized mobilization recommendations. In this context, minimally invasive stabilization may be considered in selected patients to facilitate mobilization while avoiding the morbidity of open reconstruction [11–13].

Therefore, the primary aim of this study was to compare mobility loss between geriatric patients with non- or minimally displaced acetabular fractures treated with minimally invasive screw fixation or conservative management. Secondary exploratory outcomes included medical complications, loss of autonomy, length of hospital stay, conversion to surgery, and one-year mortality.

## Materials and methods

This retrospective cohort study was conducted at a Level I trauma center and approved by the institutional ethics committee. All geriatric patients with non- or minimally displaced acetabular fractures treated between January 2015 and December 2023 were screened for eligibility. Patients were managed either with minimally invasive screw fixation or conservative treatment based on the clinical judgment of the responsible senior surgeon. Patients were assigned to the operative or conservative group according to the definitive treatment received during the index hospitalization. No standardized treatment algorithm was applied during the study period. In general, conservative treatment was preferred for stable fracture patterns in patients who could be mobilized adequately with analgesia, physiotherapy, and assistive devices. Operative treatment was considered in selected patients when early mobilization was limited by pain, when secondary displacement was considered a relevant concern, or when compliance with individualized mobilization recommendations was expected to be limited. Treatment allocation was therefore not randomized and reflected individualized clinical decision-making.

Inclusion criteria were aged 65 years or older and presented with a non- or minimally displaced acetabular fracture. Patients who underwent open reduction and internal fixation or presented with highly displaced fractures requiring open reconstruction were excluded. Patients with additional injuries that significantly impaired postoperative mobilization were also excluded.

Fractures were classified according to the Letournel and Judet classification system [14]. For statistical analysis, fracture types were grouped into simple (Letournel and Judet 1–5) and complex fracture (Letournel and Judet 6–10) patterns. Concomitant posterior pelvic ring injuries, particularly sacral fractures, were reassessed on all available CT imaging. As the classical Fragility Fracture of the Pelvis (FFP) classification requires combined anterior and posterior ring involvement and was therefore not fully applicable to the injury patterns studied, posterior pelvic ring involvement was recorded as a separate variable (yes/no) for further analysis [15].

Operative treatment consisted of minimally invasive percutaneous screw fixation performed using intraoperative navigation and three-dimensional imaging. All procedures were carried out in a hybrid operating room equipped with a floor-mounted robotic 3D C-arm (Artis Pheno, Siemens Healthineers, Forchheim, Germany) and an optical navigation system (Brainlab, Munich, Germany). In patients with concomitant posterior pelvic ring injuries, operative treatment included stabilization of both the acetabular fracture and the posterior ring. Patients undergoing operative treatment were generally mobilized with weight-bearing as tolerated using assistive devices immediately after surgery. No patient in the operative group received a formal postoperative non-weight-bearing protocol.

Conservative treatment consisted of analgesia, physiotherapy, and early mobilization using assistive devices. In most cases, patients were encouraged to mobilize with weight-bearing as tolerated. Temporary weight-bearing restrictions were recommended only in selected cases at the discretion of the treating surgeon, mainly when fracture stability, pain, or patient-specific factors raised concerns regarding unrestricted mobilization. No standardized weight-bearing protocol was applied in the conservative group during the study period.

### Outcome measures and data collection

Clinical data were extracted from electronic medical records and included patient-related variables such as age, sex, Charlson Comorbidity Index (CCI), presence of dementia, pre-injury living situation, pre-injury mobility, and fracture classification. Mechanism of injury was not included in the analysis because it was not consistently available in a standardized format across the full study period. Although information on trauma mechanism was sometimes documented in emergency department notes or admission reports, it was not reliably captured as a structured variable in the institutional data sources used for this retrospective analysis. An institutional trauma registry was not available with sufficient completeness for all included patients and the entire study period. Hospital-related parameters included length of hospital stay, discharge destination, postoperative complications, conversion to surgery, follow-up duration, and mortality.

The primary outcome was in-hospital mobility loss, defined as the difference between pre-injury Charité Mobility Index (CHARMI) and the best CHARMI score achieved during hospitalization. A higher CHARMI difference indicates greater mobility loss. Secondary exploratory outcomes included in-hospital medical complications, loss of autonomy, length of hospital stay, conversion to surgery, and one-year mortality. Loss of autonomy was defined as a new permanent admission to a nursing home in patients who had lived independently prior to injury.

### Statistical analysis

Continuous variables are presented as mean ± standard deviation and categorical variables as absolute numbers and percentages. Group comparisons were performed using Welch’s t-test for continuous variables and Fisher’s exact test for categorical variables. Mean differences or odds ratios with 95% confidence intervals were calculated as appropriate.

The primary outcome was in-hospital mobility loss and was analyzed using multivariable linear regression. Covariates were selected a priori based on clinical relevance and included age, Charlson Comorbidity Index, dementia, fracture complexity, pre-injury mobility, and posterior pelvic ring involvement. No automated variable selection procedure was used. Secondary exploratory outcomes were analyzed using the same covariate set where applicable, using logistic regression for binary outcomes and linear regression for continuous outcomes.

Model fit was assessed using the Hosmer–Lemeshow goodness-of-fit test for logistic regression models and residual diagnostics for linear regression models. Secondary outcomes were considered exploratory; therefore, no adjustment for multiple testing was performed, and* p*-values should be interpreted descriptively. A two-sided* p*-value < 0.05 was considered statistically significant for the primary analysis.

## Results

During the study period, 261 patients aged 65 years or older with acetabular fractures were screened for eligibility. Of these, 75 patients were excluded because of highly displaced fractures requiring open reduction and internal fixation, 20 patients because of severe additional injuries like traumatic brain injuries, 12 patients died of severe injuries before surgical treatment or mobilization, and 3 because of incomplete records. The final study cohort consisted of 151 patients, including 92 treated with minimally invasive screw fixation and 59 treated conservatively. Baseline characteristics of both groups are presented in Table 1. The mean age was 73.4 ± 11.9 years in the operative group and 72.2 ± 10.6 years in the conservative group. Charlson Comorbidity Index, prevalence of dementia, fracture complexity, and pre-injury mobility were comparable between groups. The distribution of fracture patterns was heterogeneous, with no single predominant fracture type. Concomitant posterior pelvic ring injuries were identified in 22 patients (14.6%). Specifically, unilateral sacral fractures were present in 14 patients (15.2%) in the operative group and in 8 patients (13.6%) in the conservative group, with no significant difference between groups (*p* = 0.99). No bilateral sacral fractures or combined anterior pelvic ring injuries were observed.

**Table 1:** Demographic data

Variable	Operative	Conservative	*p*-value
Patients (n)	92	59	
Mean age (years, SD)	73.4 ± 11.9	72.2 ± 10.6	0.500
Male (%)	56 (60.9%)	27 (45.8%)	0.093
Female (%)	36 (39.1%)	32 (54.2%)	0.419
Mean CCI (SD)	3.85 ± 2.72	3.46 ± 2.49	0.366
Dementia, n (%)	7 (7.6%)	10 (16.9%)	0.112
Follow-up, months (SD)	15.6 ± 17.7	16.8 ± 19.0	0.702
Pre-injury CHARMI (SD)	9.25 ± 1.40	9.37 ± 1.27	0.578
*Fracture type*			
Simple, n (Letournel 1–5, %)	50 (54.3%)	36 (61.0%)	0.483
Complex, n (Letournel 6–10, %)	42 (45.7%)	29 (29.0%)	0.512
Sacral fracture, n (%)	23 (25.0%)	16 (27.1%)	0.99
x (Letournel 6–10)	42 (45.7%)	23 (39.0%)	0.501

In the operative cohort, surgical complications occurred in 13 of 92 patients (14.1%). Revision surgery was required in five patients (5.4%), including two cases of postoperative hematoma requiring surgical evacuation and one case of superficial surgical site infection. In addition, two patients required secondary hip arthroplasty due to secondary fracture displacement. The underlying fracture patterns included one transverse and one T-type fracture. No neurovascular injuries or deep infections were observed. No patient in the operative group received a formal postoperative non-weight-bearing protocol. Patients were generally mobilized with weight-bearing as tolerated using assistive devices.

In the conservative group, two patients required conversion to open reduction and internal fixation due to secondary displacement where screw fixation was no longer possible. An additional three patients underwent hip arthroplasty following secondary fracture displacement. In some cases, conservative management was initially attempted, but treatment was converted to operative fixation during the same hospitalization, typically due to insufficient mobilization or persistent pain. The exact timing of conversion was not consistently documented, and no delayed conversion requiring readmission was observed.

Patients underwent regular clinical and radiographic follow-up, and clinically relevant loss of reduction was captured through the need for secondary surgical intervention, although no standardized radiographic assessment protocol was applied.

The primary outcome was in-hospital mobility loss, assessed as the difference between pre-injury CHARMI and the best CHARMI score achieved during hospitalization. In unadjusted analysis, operatively treated patients showed less in-hospital mobility loss than conservatively treated patients. The mean CHARMI difference was 2.53 ± 1.73 in the operative group compared with 3.44 ± 1.57 in the conservative group, corresponding to a mean difference of − 0.91 points (95% CI − 1.45 to − 0.37; *p* = 0.0011). However, after adjustment for age, Charlson Comorbidity Index, dementia, fracture complexity, pre-injury mobility, and posterior pelvic ring involvement, treatment modality was not independently associated with in-hospital mobility loss (β − 0.07, 95% CI − 0.59 to 0.44; *p* = 0.780).

Secondary exploratory outcomes are summarized in Table 2. Among patients who had lived independently prior to injury, loss of autonomy occurred in 20 patients (21.7%) in the operative group and in 22 patients (37.2%) in the conservative group; this difference did not reach statistical significance (OR 0.49, 95% CI 0.23–1.02; *p* = 0.062).

No significant difference in medical complications between the two cohorts was found (*p* = 0.211). 25 out of 92 (27.2%) patients suffered from one or more non surgical complication in the operative cohort such as urinary tract infection (14.1%), pneumonia (7.6%), anaemia (3.3%) or cardial decompensation (2.2%). In the conservative group a total of 22 medical complications (37.2%) were found. Eight patients suffered from urinary tract infection (13.6%), five from pneumonia (8.5%) and three from cardiac decompensation (5.1%). The other patients had complications like hyponatremia, hypokalemia, urolithiasis and anaemia (8.5%).

One-year mortality was significantly lower in the operative group compared with the conservative group (10.9% vs. 30.5%; OR 0.28, 95% CI 0.12–0.66; *p* = 0.0046). However, after multivariable adjustment, treatment modality was not independently associated with one-year mortality (aOR 0.40, 95% CI 0.13–1.19; *p* = 0.098).

Length of hospital stay did not differ significantly between groups, with a mean duration of 16.1 ± 7.9 days in the operative group and 17.2 ± 7.9 days in the conservative group.

**Table 2:** Outcome parameters

Outcome	Operative	Conservative	Effect size (95% CI)	*p*-value
Surgical complications (n, %)	13 (14.1%)			
Revision surgery (n, %)	5 (5.4%)			
Conversion to ORIF (n, %)		2 (3.4%)		
Conversion to Arthroplasty (n, %)		3 (5.1%)		
Medical complications (n, %)*	25 (27.2%)	22 (37.3%)	OR 0.63 (0.31–1.26)	0.211
1 year mortality (n, %)*	10 (10.9%)	18 (30.5%)	**OR 0.28 (0.12–0.66)**	**0.0046**
Loss of autonomy (n, %)*	20 (21.7%)	22 (37.2%)	OR 0.49 (0.23–1.02)	0.062
CHARMI difference (mean, SD) †	2.53 ± 1.73	3.44 ± 1.57	**−0.91 (− 1.45 to − 0.37)**	**0.0011**
Length of stay (d, SD) †	16.1 ± 7.9	17.2 ± 7.9	−1.13 (− 3.73 to 1.48)	0.393
Letournel 6–10)	42 (45.7%)	23 (39.0%)	0.501	

*Fishers exact test, †Welch’s t-test

Exploratory multivariable regression analyses adjusting for age, Charlson Comorbidity Index, dementia, fracture complexity, pre-injury mobility and posterior ring involvement are presented in Table 3. In these adjusted models, treatment modality was not independently associated with loss of autonomy, medical complications, mortality, mobility loss, or length of hospital stay. Model-fit assessment did not indicate relevant lack of fit for the regression models.

**Table 3:** Multivariable regression analyses adjusted for age, Charlson Comorbidity Index, dementia, fracture complexity, and pre-injury mobility and posterior pelvic ring involvement

Outcome	Adjusted treatment effect	*p*-value
Medical complications *	aOR 0.57 (0.27–1.18)	0.129
1 year mortality *	aOR 0.40 (0.13–1.19)	0.098
Loss of autonomy *	aOR 0.77 (0.34–1.76)	0.534
CHARMI difference †	β − 0.07 (− 0.59 to 0.44)	0.780
Length of stay †	β − 0.68 (− 3.36 to 2.01)	0.618
	42 (45.7%)	23 (39.0%)

*Multivariable logistic regression †Multivariable linear regression

### Case

**Fig. 1:**
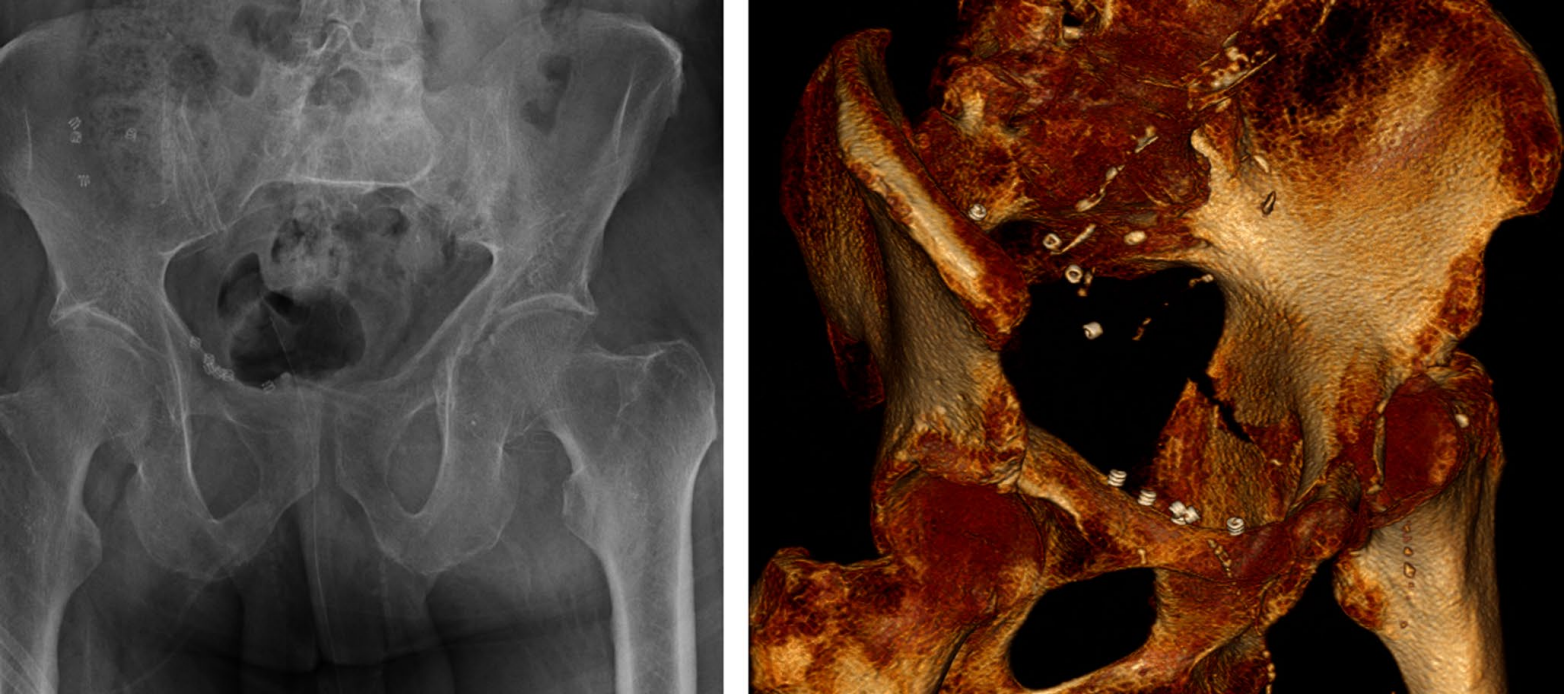
86 year old Patient with transverse fracture of the left acetabulum

An 86-year-old patient was referred to us after falling at home. Diagnostics revealed a transverse fracture according to Letournel and Judet as well as an accompanying unilateral sacral fracture (Fig. 1).

**Fig. 2:**
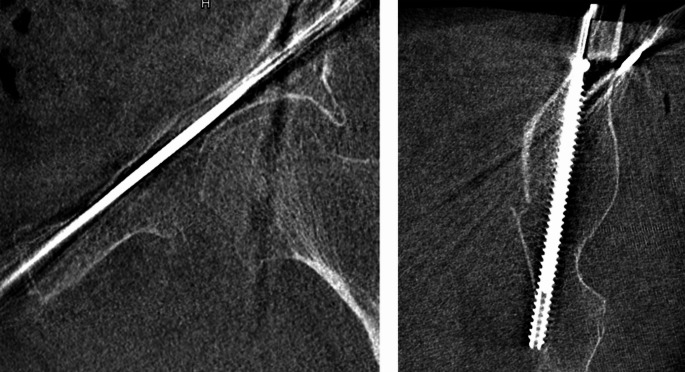
Insertion of the navigated guide-wires as well as the screws in the anterior column (left) and the posterior column (right)

**Fig. 3:**
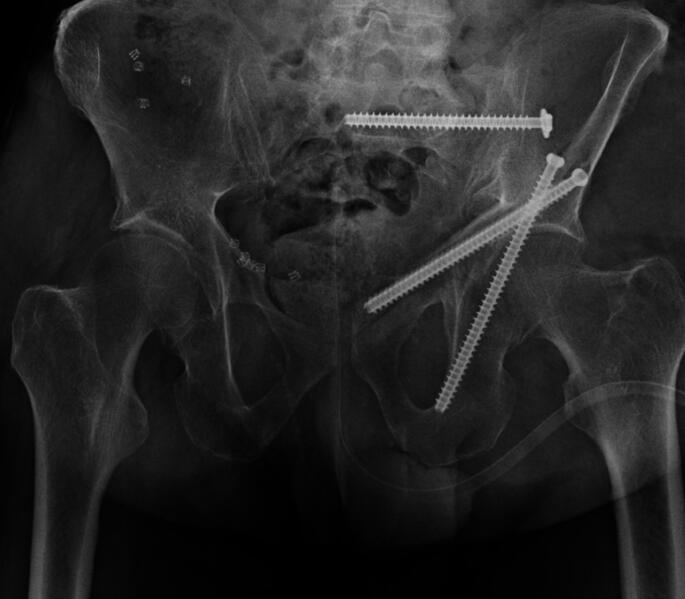
6 week follow-up

Based on the fracture morphology, minimally invasive surgery was performed to facilitate early mobilization and pain relief. After preparation and planning of the screws, the navigated guide wires were inserted (Fig. 2). After 3D verification of the wires, the 7.3 mm cannulated screws were implanted (Fig. 3). The patient was mobilized during the course of treatment and was discharged to follow-up rehabilitation.

## Discussion

The present study evaluated clinical outcomes of minimally invasive screw fixation compared with conservative treatment in geriatric patients with non- or minimally displaced acetabular fractures. In unadjusted analysis, operative treatment was associated with less in-hospital mobility loss. However, this association was not confirmed after adjustment for baseline mobility and relevant confounders. Similarly, unadjusted differences in one-year mortality were not confirmed in the predefined multivariable model. These findings suggest that the observed differences between treatment groups were strongly influenced by baseline patient characteristics and treatment selection.

The management of acetabular fractures in geriatric patients remains challenging. Increasing life expectancy and the growing prevalence of osteoporosis have led to a rising incidence of fragility fractures of the pelvis and acetabulum [3, 13]. In this patient population, prolonged immobilization is associated with substantial morbidity, including pneumonia, thromboembolic events, muscle wasting, and functional decline. Consequently, treatment strategies that allow early mobilization have gained increasing attention in recent years [7, 16].

Minimally invasive screw fixation represents a potential alternative to conservative treatment in selected fracture patterns. Percutaneous stabilization allows mechanical fracture fixation while minimizing surgical trauma and soft-tissue damage compared with open reduction and internal fixation [17, 18]. Biomechanical studies even suggest that there is no significant difference in the stability of plate osteosynthesis compared to minimally invasive screw osteosynthesis regarding the dislocation rate under load [19]. In the present cohort, surgical complications occurred in 14.1% of operatively treated patients, and revision surgery was required in 5.4% of cases. Importantly, no neurovascular injuries or deep infections were observed, suggesting that the technique can be performed safely when appropriate imaging and surgical expertise are available.

In-hospital mobility remains a clinically relevant outcome in geriatric trauma patients, as functional decline during hospitalization is associated with loss of independence and adverse long-term outcomes [20, 21]. Although operatively treated patients showed less mobility loss in the unadjusted analysis, this association was not maintained after adjustment. Therefore, the observed difference should not be interpreted as evidence of a causal treatment effect, but rather as a finding that may reflect both treatment effects and selection of patients considered suitable for surgery. In addition, weight-bearing recommendations require consideration when interpreting mobility outcomes. In the present cohort, most conservatively treated patients were encouraged to mobilize with weight-bearing as tolerated using assistive devices, while temporary restrictions were recommended only in selected cases. This is in line with other reports supporting nonoperative treatment with early weight-bearing as tolerated in selected elderly patients with minimally displaced acetabular fractures [22–24].

Similarly, one-year mortality was substantially lower in the operative group compared with the conservative group. Although causality cannot be inferred from the present retrospective design, this finding may be influenced by differences in baseline characteristics and treatment selection, as reflected by the loss of significance after multivariable adjustment. Previous studies have demonstrated that prolonged immobilization and delayed mobilization are important predictors of adverse outcomes in elderly trauma patients [20, 21, 25, 26].

Loss of autonomy represents another clinically important outcome in geriatric trauma care [27–31]. In the present study, fewer operatively treated patients required new admission to a nursing home compared with conservatively treated patients. Although this difference did not reach statistical significance, the observed trend suggests that improved mobility and fracture stability may help preserve independence in selected patients.

When potential confounding factors were considered in multivariable regression analyses, treatment modality was no longer independently associated with the investigated outcomes. This highlights the strong influence of baseline patient characteristics and potential selection bias. In particular, the discrepancy between unadjusted and adjusted mortality analyses suggests that the observed mortality difference may reflect residual confounding rather than a causal treatment effect.

This study has several limitations. The retrospective design and individualized treatment decisions introduce a risk of selection bias. In addition, the single-center setting, imaging infrastructure, and surgical expertise required for percutaneous acetabular fixation may limit generalizability. In our cohort, all procedures were performed in a hybrid operating room; however, the technique itself does not depend on a hybrid suite and can also be performed with mobile intraoperative 3D imaging and navigation. Still, these resources and the required experience are not available in all centers, which may limit transferability of the results. Although the cohort size was relatively large for this fracture pattern, statistical power remains limited. Functional outcomes were assessed using in-hospital mobility rather than long-term patient-reported measures. In addition, the impact of posterior pelvic ring injuries cannot be fully excluded, and length of hospital stay reflects differences in healthcare systems. The mechanism of injury was not consistently documented and could not be included in the analysis. This may represent an additional source of confounding, as high-energy trauma in otherwise fit elderly patients may influence both treatment allocation and outcomes.

Despite these limitations, the present study provides clinically relevant insights into the management of geriatric acetabular fractures. The results suggest that minimally invasive screw fixation may represent a useful treatment option for selected patients with non- or minimally displaced acetabular fractures, particularly when early mobilization is a primary treatment goal.

## Conclusion

In this retrospective cohort of geriatric patients with non- or minimally displaced acetabular fractures, minimally invasive screw fixation was associated with less in-hospital mobility loss in unadjusted analysis, but this association was not confirmed after adjustment for baseline mobility and other confounders. Secondary outcomes, including one-year mortality, should be interpreted as exploratory. Minimally invasive fixation may be considered in selected patients who fail to mobilize adequately under conservative treatment, but no causal conclusion regarding superiority over conservative management can be drawn.

## Data Availability

No datasets were generated or analysed during the current study.
